# Joint Modelling of Survival and Emergency Medical Care Usage in Spanish Insureds Aged 65+

**DOI:** 10.1371/journal.pone.0153234

**Published:** 2016-04-13

**Authors:** Xavier Piulachs, Ramon Alemany, Montserrat Guillen

**Affiliations:** Department of Econometrics, Riskcenter-IREA, University of Barcelona, Barcelona, Spain; University at Albany State University of New York, UNITED STATES

## Abstract

**Background:**

We study the longevity and medical resource usage of a large sample of insureds aged 65 years or older drawn from a large health insurance dataset. Yearly counts of each subject's emergency room and ambulance service use and hospital admissions are made. Occurrence of mortality is also monitored. The study aims to capture the simultaneous dependence between their demand for healthcare and survival.

**Methods:**

We demonstrate the benefits of taking a joint approach to modelling longitudinal and survival processes by using a large dataset from a Spanish medical mutual company. This contains historical insurance information for 39,137 policyholders aged 65+ (39.5% men and 60.5% women) across the eight-year window of the study. The joint model proposed incorporates information on longitudinal demand for care in a weighted cumulative effect that places greater emphasis on more recent than on past service demand.

**Results:**

A strong significant and positive relationship between the exponentially weighted demand for emergency, ambulance and hospital services is found with risk of death (alpha = 1.462, *p* < 0.001). Alternative weighting specifications are tested, but in all cases they show that a joint approach indicates a close connection between health care demand and time-to-death. Additionally, the model allows us to predict individual survival curves dynamically as new information on demand for services becomes known.

**Conclusions:**

The joint model fitted demonstrates the utility of analysing demand for medical services and survival simultaneously. Likewise, it allows the personalized prediction of survival in advanced age subjects.

## Introduction

Rising rates of longevity are closely tied to the growth in demand for medical attention and long-term care services, the increasing usage of which extends longevity even further. Joint models of longitudinal and survival data enable us to estimate the simultaneous association between survival and the demand for care, and serve as a tool for predicting risk of death based on personalized medical records. Thus, as an individual’s risk of death increases, the probability of their being hospitalized increases and having been admitted to hospital, their probability of death is also greater than that of a non-hospitalized individual.

The aim of this paper is to examine the use of joint modelling techniques for predicting survival. Our motivating dataset includes 39,137 Spanish insureds aged 65 and over, on which we conducted a longitudinal and time-to-death monitoring study from 1 January 2006 to 1 February 2014. Whereas classical survival prediction is based solely on patients’ past medical records [1,2], joint modelling has the advantage that intensive care usage and survival are modelled simultaneously [3–6], by assuming a zero-mean latent Gaussian process that correlate the two settings [7–9]. Moreover, recent and current records, as opposed to information about medical usage that occurred many years ago, have a greater incidence on an individual’s current health status, suggesting that information should be weighted with an appropriate decreasing function. Here, we investigate the role of the information contained in medical records and identify a *fading* effect, whereby more recent records have a greater influence than older medical records on the risk of death.

Medical emergency claim counts serve as a measure of annual medical care usage and, as such, of a patient's health status. Changes in these biomarker counts allow us to make dynamic subject-specific predictions, that is, personalized survival curves can be systematically updated as new measurements are collected. Thus, the joint modelling scheme proposed here is a powerful, dynamic tool, especially applicable to subjects afflicted by chronic processes [10–12]. In summary, joint models allow us to: (i) estimate both longitudinal and survival parameters without the bias introduced by a separate analysis; (ii) confirm that age and gender affect survival changes, even when controlling for hospitalization and emergency care unit visits; (iii) test the significance of the degree of association between care usage and survival; (iv) estimate subject-specific survival probabilities; and (v) update personalized survival estimations as additional longitudinal data are collected.

## Methods

### Health insurance dataset

Our study examines a large dataset provided by a Spanish medical mutual company, from which we collected a random sample of policyholders aged 65 and above. All patient records/information was anonymized and de-identified prior to analysis. The UB Riskcenter’s ethics committee approved this retrospective study.

The data contain historical information on claims reported between 1 January 2006 and 1 February 2014 by 39,137 policyholders (aged 65+), 39.5% of whom were men and 60.5% women. The time of origin for each policyholder's record is taken as their date of entry into the study (*t* = 0), and from that point on all subjects continued to be monitored until the end of the study period. The dataset provides reports on all the claims made by each policyholder in eight consecutive one-year windows. As our primary interest lies in non-routine care, usage counts only include hospitalizations, emergency service visits and the use of ambulance services. The data can be consulted at www.ub.edu/riskcenter/R/jm.

The mean age of policyholders entering the study is 73.2 yr for men and 74.2 yr for women. It is well documented that women have a longer average life expectancy and worse health status [13–14]. Data presented in Table 1 show the frequencies and row percentages for age-at-entry groups and by gender. Observed deaths are also presented. In the 65-to-74 age-at-entry group the number of deaths is higher for women than for men (261 vs. 207), but as the number of insured women (13,121) is much higher than the number of insured men (9,370), the death rate is lower for the former (2.0% vs. 2.2%).

**Table 1:** Description of policyholders, deaths and death rate distributions by age-at-entry and gender. Number of subjects (frequencies and row percentages), deaths occurring prior to study completion (frequencies and row percentages) and percentage death rates are stratified by age-at-entry and gender.

Gender	Age at entry (years)			
	[65,75)	[75,85)	≥85	Overall
Total (%)	22491 (57.5)	13066 (33.4)	3580 (9.1)	39173 (100.0)
Men (%)	9370 (60.6)	4937 (31.9)	1154 (7.5)	15461 (100.0)
Women (%)	13121 (55.4)	8129 (34.3)	2426 (10.3)	23676 (100.0)
Deaths (%)	468 (15.0)	1524 (48.7)	1137 (36.3)	3129 (100.0)
Men (%)	207 (21.1)	474 (48.4)	299 (30.5)	980 (100.0)
Women (%)	261 (12.1)	1050 (48.9)	838 (39.0)	2149 (100.0)
Percent death rate	2.1	11.7	31.8	8.0
Men (%)	2.2	9.6	25.9	6.3
Women (%)	2.0	12.9	34.5	9.1

Overall, the date of death was recorded for 3,129 individuals during the follow-up period, so that 92.0% of policyholders survived or were no longer in the sample at the end of the study. Although policyholders aged over 85 represented just 9.1% of the dataset, they accounted for 36.3% of deaths. As for the actual length of the follow-up, almost half of all policyholders were included throughout the observation window. As such, some of the right-censored profiles result from the administrative closure date, with some policyholders having left the insurance company. The censoring mechanism, other than that attributable to the end of the study, can therefore be assumed to be unrelated to the death-event, as it refers to insurance cancellations attributable to reasons of an external nature (primarily dissatisfaction with the service, a change of company or an unwillingness to pay).

We consider the demand for health claims in the year preceding the observation point as our longitudinal outcome, that is, the number of times a subject requests hospital, emergency room or ambulance services. This was introduced in the model using a logarithmic scale to allow for a log-linear specification [15]. In the survival approach, the baseline covariates are *age0* (age at entry) and *sex* (man:0; woman:1). The former plays an essential role by providing a suitable survival characterization for *late* entries, i.e. policyholders who enter the study after the age 65, whereas the *sex* covariate allows us to distinguish different patterns of behaviour by gender.

### Longitudinal approach

The observed longitudinal response included in the model summarizes a policyholder’s past demand for hospital and emergency services and corresponds to an accumulation of past claims. Such observed data are transformed as the logarithm of one plus the number of services demanded during the year prior to the observation point. This is denoted by *y*_*ij*_ = {*y*_*i*_*(t*_*ij*_*)*,*j* = 1,…,*n*_*i*_}, where *i* is the individual and *n*_*i*_ is the number of measurements made of a particular person. The specific occasions when the policyholder is measured are denoted by *t*_*ij*_. A standard log-linear mixed model is used to explain the mathematical expectation of service usage, which we denote by *m*_*i*_*(t)*. A Gaussian approximation is assumed in the response [16].

In order to give greater weight to more recent claims than to older claims, F(·) is defined as the cumulative weighted area under the claim's historical path. Since we assume that the historical effects fade over time, we define

$$F\left(t\right)=\int \bar{w}\left(t-s\right)\;m\left(s\right)\text{ d}s\;,\;s<t$$ (1)

where $\overset{-}{w}(\cdot )$ is the weighting function [17,18]. Alternative weightings for capturing the *fading* effect of service demand on survival were tested. For the random effects structure, we firstly considered a random intercept model (a single random effect). Additionally, a random slope effect via a bivariate random effects structure has been also tested.

### Survival approach

Given our particular interest in discerning gender survival differences among elderly policyholders, it is highly informative to know the distribution of the true event times for the *i*-th policyholder, $T_{i}{}^{*}$, defined in our study as a non-negative random variable that includes the time lag from the point a policyholder enters the study until their death-event. This approach involves the probabilistic distribution of the timing of death, considering the proportion of living subjects beyond time point *t* via the corresponding survival function,*S*(*t*) = Pr (T* *>* *t*). A corresponding potential right-censored time *C*_*i*_ is assumed since some policyholders may still be alive when their coverage terminates, so that in practice we can only observe event times,$\;T_{i}=\text{min }\left\{T_{i}{}^{*},C_{i}\right\}$.

Under the above assumptions, the Cox proportional hazards model [19] can be defined as:

$$h_{i}\left(t\;|\;w_{i}\right)=h_{0}\left(t\right)\text{ exp }\left\{\gamma^{\text{T}}w_{i}\right\},\text{ conditional on }t>0,$$ (2)

where *h*_0_(*t*) is the baseline risk function, ***w***_*i*_ is the vector of baseline survival covariates and ***γ*** the vector that contains the corresponding regression parameters. Complete information of this nature can only be known at fixed time points. Although *h*_0_(·) remains traditionally left unspecified, this condition was relaxed so that subject-specific predictions could be made more easily. Thus, the logarithm of the baseline function was approached using a cubic *B*-spline basis function [20].

### Joint model with recency-weighted cumulative effects

The joint model is specified as both a longitudinal and a survival process simultaneously, where *α* is the parameter that captures the effect of the policyholder’s background health status on her survival. Therefore, the joint model can be defined as

$$\{\begin{array}{l} y_{i}\left(t\right)=m_{i}\left(t\right)+\varepsilon_{i}\left(t\right)\\ \;m_{i}\left(t\right)=x_{i}{}^{\text{T}}\left(t\right)\beta +z_{i}{\left(t\right)}^{\text{T}}b_{i}\\ \begin{array}{l} \;b_{i}\;\sim \;N\left(0,D\right)\;,\;\varepsilon_{i}\left(t\right)\;\sim \;N\left(0,\sigma^{2}\right)\\ h_{i}\left(t\;|\;M_{i}\left(t\right),\;w_{i}\right)=h_{0}\left(t\right)\text{ exp}\left\{\gamma^{\text{T}}w_{i}+\alpha \;F\left(m_{i}\left(t\right)\right)\right\}\;, \end{array} \end{array}$$ (3)

where $M_{i}(t)\;=\;\{{\;m}_{i}(s),\;0\leq s\leq t\}$ denotes the complete policyholder-specific history of the expected longitudinal response until time t and $F(m_{i}(t))$ are the recency-weighted cumulative effects of the longitudinal process. In practice, this captures the influence of past claims on the survival model. Moreover, for the intercept and slope random effects, ***b***_*i*_ = (*b*_*i*0_, *b*_*i*1_)^T^, a bivariate normal distribution with zero mean and unknown unstructured variance-covariance matrix ***D*** is assumed, where $d_{11}\;=\;Var(b_{i0})\;=\;\sigma_{b0}^{2},\;d_{22}\;=\;Var(b_{i1})\;=\;\sigma_{b1}^{2}$ and *d*_12_ = Cov(*b*_*i*0_, *b*_*i*1_) = *ρ*_*b*0*b*1_*σ*_*b*0_*σ*_*b*1_. The Akaike Information Criterion (AIC) was used as a global goodness-of-fit measure and its value served as the basis for ranking the fitted models. The lowest AIC value corresponded to exponentially weighted records, which points to the marked impact of medical services used in the last year of life (accounting for the overall weight) on the death hazard rate.

All analyses were performed with R statistical software (www.rproject.org) version 3.2.2 [21], using the JM package [22]. Moreover, to compare the prediction performance we also used JMbayes package [23] to implement the Bayesian approach.

### Individualized survival predictions

One of the key features of joint modelling techniques is that personalized predictions for the time-to-event outcome can be obtained from a consideration of all subject-specific longitudinal measurements. Joint modelling means that longitudinal outcomes preceding present time *t* are directly related to current mortality probability as well as to the probability of surviving at a later time *u* conditional on being alive at *t*. As such, an accurate knowledge of a subject's past health problems is essential for providing personalized survival predictions.

Let us consider a generic policyholder aged over 65 not included in the original dataset $D_{n}\;=\;\left\{{\;T}_{i},\;{\;\delta }_{i},{\;y}_{i}(t),\;i\;=\;1,\ldots ,n\;\right\},\;$ but within the target population. This individual has a historical set of emergency medical care records ${\;Y}_{k}(t)\;=\;\{y(s);0\leq s\leq t\}$ as well as a particular vector of survival baseline covariates ***w***_*k*_. Thus, the prognosis task relies on estimating the survival probability according to the joint generalized linear model and subject-specific information. Let us assume that the subject is alive at time *t*, then our primary objective is to estimate the probability of her remaining alive at any future time *u* > *t*,

$$\pi_{k}\left(u\;|\;t\right)=\text{Pr }\left(T^{*}{}_{k}\geq u\;|\;T^{*}{}_{k}>t,\;Y_{k}\left(t\right),\;w_{k},\;D_{n},\;\theta \right).$$ (4)

By adopting a Bayesian strategy, the above expression can be estimated from the corresponding posterior as

$$\;\pi_{k}\left(u\;|\;t\right)=\int_{\theta }\text{Pr }\left(T^{*}{}_{k}\geq u\;|\;T^{*}{}_{k}>t,\;Y_{k}\left(t\right),\;D_{n}\right)p\left(\theta \;|\;D_{n}\right)\text{ d}\theta$$ (5)

where the first term of the above integral is obtained through conditional independence between the longitudinal and survival responses, and the posterior distribution $p(\theta \;|{\;D}_{n})$ is derived from the asymptotic approximation $\;\theta \;|{\;D}_{n}\;\sim \;N(\hat{\theta },\;V(\hat{\theta }))\;$ via Markov chain Monte Carlo (MCMC) sampling, where $\hat{\theta }\;$ is the maximum likelihood estimate for the *true* parameter vector ***θ***. Finally, a Monte Carlo estimate of *π*_*k*_(*u*|*t*)is obtained by combining the previous assumptions.

## Results

We first present the estimation obtained when applying the joint model and compare these results with those obtained under a separate estimation of the longitudinal and survival sub-models that ignores the dependence between these components for each subject. The maximum likelihood estimates of the main parameters from both strategies are presented in Table 2.

**Table 2:** Comparison of the estimates obtained from the separate analyses and from the joint model. Parameter estimates for the models applied to the health insurance dataset (n° of cases = 39,173). The longitudinal process captures the log-transformed demand for hospitalization, emergency room care and ambulance services in the year preceding observation and the event process (defined by an exponential weighting function) models survival.

Approach	Parameter	Estimate	Std. Err.	95% Conf. Int.	*p*‒value
Separate analyses	Longitudinal Process				
	*β*_0_	0.320	0.003	(0.315, 0.325)	< 0.001
	*β*_1_	0.013	0.001	(0.012, 0.014)	< 0.001
	*σ*_*b*0_	0.325	0.003	(0.320, 0.330)	
	*σ*_*b*1_	0.050	0.001	(0.048, 0.051)	
	*σ*	0.472	0.001	(0.470, 0.473)	
	Event Process				
	*γ*_*sex*(*woman*)_	– 0.722	0.426	(– 1.556, 0.112)	0.090
	*γ*_*age*0_	0.144	0.004	(0.136, 0.152)	< 0.001
	*γ*_*sex*(*woman*)×*age*0_	0.010	0.005	(0.000, 0.021)	0.048
Joint Model (Frequentist)	Longitudinal Process				
	*β*_0_	0.319	0.003	(0.314, 0.324)	< 0.001
	*β*_1_	0.014	0.001	(0.013, 0.015)	< 0.001
	*σ*_*b*0_	0.329	0.003	(0.323, 0.334)	
	*σ*_*b*1_	0.050	0.001	(0.048, 0.051)	
	*σ*	0.471	0.001	(0.469, 0.473)	
	Event Process				
	*γ*_*sex*(*woman*)_	– 4.648	0.427	(– 5.484, – 3.811)	< 0.001
	*γ*_*age*0_	0.095	0.004	(0.086, 0.103)	< 0.001
	*γ*_*sex*(*woman*)×*age*0_	0.059	0.005	(0.049, 0.070)	< 0.001
	Association				
	*α*	1.462	0.057	(1.345, 1.574)	< 0.001

The random effects in the longitudinal mixed model provide robust estimations for both separate and joint approach, with very little difference in the parameter estimates or in their corresponding standard errors. The estimates of the population-averaged parameters ${\hat{\beta }}_{0}$ and ${\hat{\beta }}_{1}$ are both positive and describe an overall upward trend. In the case of the survival sub-model, in contrast, a difference in the estimation is detected in the case of the joint model specification. A marked improvement is also recorded in the significance of the baseline survival parameters, as indicated by the smaller *p*-values, and the readjustment of the estimated coefficients. However, the main result is the presence of a significant association between longitudinal demand and the event outcome, since parameter *α* is strongly significant and positive, *α* = 1.462 (*p*-value < 0.001). Thus, we infer an increasing relationship between the frequency of use of non-routine medical services and the risk of death. For the random effects structure, we firstly considered a random-intercept model (a single random effect). However, the posterior inclusion of a random slope effect indicated that the rate of change in the claiming evolution was significantly different form policyholder to policyholder, as reflected by the likelihood ratio test (p-value < 0.001).

**Fig 1:**
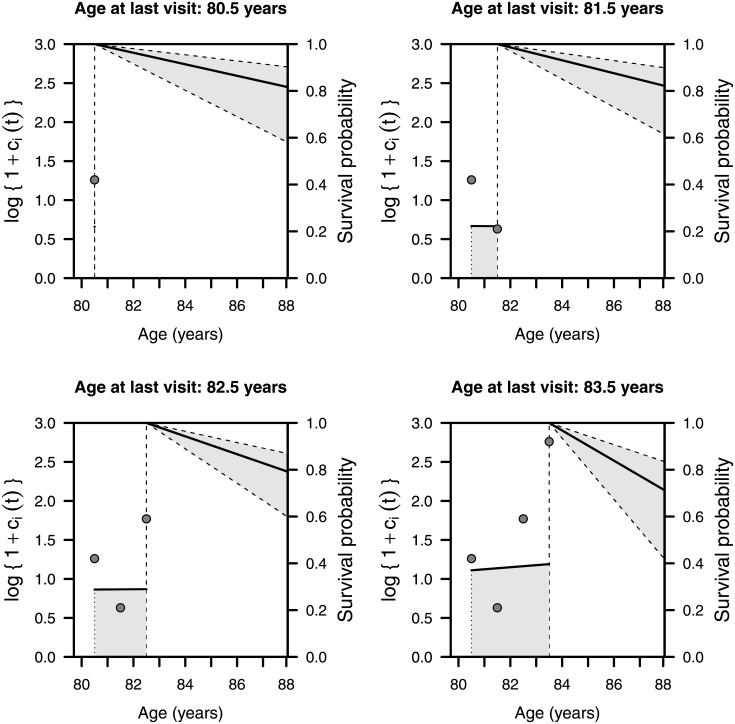
Subject-specific longitudinal evolution of weighted cumulative exposure and dynamic survival probability for a woman aged 80 at study entry. The plot is distributed in four panels showing the first four successive measuring points, at ages 80.5, 81.5, 82.5 and 83.5 years, respectively. The left-hand side of each panel depicts the cumulative area under the true biomarker path until the time of measurement, while the right-hand side shows the median predicted survival probabilities over 200 Monte Carlo samples. The shaded region in the survival estimate is limited by the 95% pointwise confidence intervals.

As for the structure of the joint model, we assume that an integrated measure of the claims history data is the most appropriate way to incorporate the claims data "biomarker" in the joint modelling framework. Although this appears reasonable, we would also expect the rate of rise in the actual claims history, e.g. the number of new claims made in a given period of time, to also provide information. Policyholders presenting a more rapid rise in their claims process are likely to be in poorer health, otherwise they would not be seeking medical attention. Thus, this measure appears to account for the "fading" behaviour captured by recency-weighted information on claims. We have explored the sensitivity of these inferences to the derivative (slope) of the claims history process, further improvement is obtained from extending the standard joint model parametrization (that is, by considering the current biomarker value) to the rate parametrization (*p*‐value = 0.094). In the case of policyholders with the same age at study entry, the estimated hazard ratio of death for females relative to males is exp(– 0.722) = 0.486, i.e. (1–0.486)·100 = 51.4% lower, although it is not a significant reduction parametrization (*p*-value = 0.090). On the other hand, in the joint model when considering policyholders with the same level of claims and same age at study entry, the estimated hazard ratio of death for females relative to males is exp(– 4.648) = 0.010, which is now significantly lower (*p*-value < 0.001). The four panels in Fig 1 depict an example of the annual updating at each measurement point in both the subject's biomarker evolution and her estimated survival curve. Taking the age of 88 as our threshold reference, the plots show a declining survival probability due to an increasing cumulative area under the logarithm of hospital/emergency health service demand. Thus, a more marked expansion of the shaded area reduces the probability of staying alive until the age of 88. The data structure assumes the absence of observed deaths after the given closing date, which impedes making predictions beyond that date. In spite of the short exposure considered here, the monitored period is long enough for us to infer once again a significant association between care usage and time-to-death. Note that the inclusion of new data will not only reduce the standard error of the estimates, but also extend the time horizon of the survival curves.

To illustrate the difference between the joint and separate survival models, it can be seen in Table 3 that the woman’s survival probability at age 88 is 0.790 by joint modelling techniques, but that the estimated value is 0.755 in the separate survival model and would remain the same regardless of information on current and past emergency claims. The performance of the joint model also differs from that of the separate survival model; thus, if we compare the prediction error of a joint model (at time 7, the estimated prediction error with absolute loss function is 0.064 when using information available up to time 5) with that of a separate survival model (at time 7, the corresponding estimated prediction error with absolute loss function is 0.070), we can confirm that the joint model performs better in terms of prediction because the error is smaller.

**Table 3:** Subject-specific dynamic survival probabilities for a woman aged 80 at study entry, with the claims observed in Fig 1. The mean and median estimated survival probabilities at the age of 88 for the four successive measuring points, corresponding to different ages over 200 Monte Carlo samples and the 95% pointwise confidence intervals for the mean.

Age	Mean	Median	Lower	Upper
80.5 years	0.790	0.814	0.572	0.901
81.5 years	0.799	0.818	0.606	0.897
82.5 years	0.771	0.787	0.589	0.866
83.5 years	0.682	0.704	0.400	0.829

The size of our sample is large, although small in comparison to the health claims data typical of other contexts (e.g. the US, Canada or the NHS in the UK); therefore, computational time may be an issue when implementing joint models. Here, for instance, the model estimation takes about 21 minutes on a standard PC. The Bayesian approach requires considerably more time, which is why we address Bayesian illustrations with a smaller random sample (specifically, a 10% random fraction). For this smaller sample, the estimation time for the frequentist approach is 79.1 seconds, while it is 10.2 hours for the Bayesian approach. Thus, the Bayesian approach requires much more computational time on a standard PC than is required by the frequentist approach.

We report the model estimates from the Bayesian and the frequentist approaches in Table 4, for this smaller sample of 3,915 policyholders. In particular, we performed three MCMC chains with 35,000 iterations, of which 5,000 were used for the burn-in period. The results provided by the two approaches do not vary substantially. By switching to a Bayesian approach, besides estimating the model, we also calculate numerical measures evaluated by MCMC methods (see the documentation of the JMbayes package [23]). The prediction error for the smaller sample under the Bayesian approach is 0.065 by considering an absolute loss function, which is exactly the same as that obtained under the frequentist approach for the same sample size.

**Table 4:** Comparison of the estimates obtained from the separate analyses and from the joint model with the frequentist and the Bayesian approaches. Parameter estimates are based on the small health insurance dataset (n° of cases = 3,915). The longitudinal process captures the demand for hospitalization, emergency room care and ambulance services in the year preceding observation and the event process (defined by an exponential weighting function) models survival.

Approach	Parameter	Estimate	Std. Err.	95% Conf. Int.	*p*‒value
Separate analyses	Longitudinal Process				
	*β*_0_	0.342	0.008	(0.326, 0.359)	< 0.001
	*β*_1_	0.010	0.002	(0.007, 0.010)	< 0.001
	*σ*_*b*0_	0.339	0.008	(0.323, 0.359)	
	*σ*_*b*1_	0.045	0.002	(0.040, 0.050)	
	*σ*	0.477	0.003	(0.472, 0.483)	
	Event Process				
	*γ*_*sex*(*woman*)_	– 0.347	1.430	(– 3.151, 2.456)	0.808
	*γ*_*age*0_	0.151	0.015	(0.122, 0.180)	< 0.001
	*γ*_*sex*(*woman*)×*age*0_	0.005	0.018	(– 0.029, 0.039)	0.772
Joint Model (Frequentist)	Longitudinal Process				
	*β*_0_	0.341	0.008	(0.325, 0.358)	< 0.001
	*β*_1_	0.011	0.002	(0.008, 0.014)	< 0.001
	*σ*_*b*0_	0.342	0.010	(0.324, 0.360)	
	*σ*_*b*1_	0.045	0.002	(0.041, 0.050)	
	*σ*	0.477	0.012	(0.454, 0.499)	
	Event Process				
	*γ*_*sex*(*woman*)_	– 4.516	1.376	(– 7.212, – 1.820)	0.001
	*γ*_*age*0_	0.094	0.014	(0.067, 0.122)	< 0.001
	*γ*_*sex*(*woman*)×*age*0_	0.058	0.017	(0.024, 0.092)	0.001
	Association				
	*α*	1.454	0.177	(1.107, 1.801)	< 0.001
Joint Model (Bayesian)	Longitudinal Process				
	*β*_0_	0.304	< 0.001	(0.292, 0.316)	< 0.001
	*β*_1_	0.038	< 0.001	(0.018, 0.058)	< 0.001
	*σ*_*b*0_	0.282	0.001	(0.261, 0.301)	
	*σ*_*b*1_	0.598	< 0.001	(0.584, 0.612)	
	*σ*	0.473	< 0.001	(0.467, 0.478)	
	Event Process				
	*γ*_*sex*(*woman*)_	– 2.473	0.141	(– 4.496, – 0.289)	0.024
	*γ*_*age*0_	0.121	0.003	(0.107, 0.143)	< 0.001
	*γ*_*sex*(*woman*)×*age*0_	0.035	0.002	(0.008, 0.060)	0.015
	Association				
	*α*	1.615	0.006	(1.445, 1.850)	< 0.001

## Discussion

An increasing number of statistical studies report the individualized monitoring of time-dependent covariates prior to the occurrence of a particular event. Here, we have shown that the joint analysis of the individualized and of the true longitudinal evolution over the lifetime is not only indispensable for detecting the strength of association between two responses, but additionally helps improve parameter estimates considerably by circumventing the potential risk of bias caused by the incomplete follow-up of certain subjects. The key feature of the joint approach lies in the inclusion of longitudinal information within the standard survival model by means of a latent Gaussian process, which is specifically materialized in their shared random effects purpose.

The number of events in our claims dataset is in fact low and most of the patients are actually still alive by the end of the observation period. This is the most likely explanation for the numerical identity of the coefficients of the longitudinal process, when modelled both separately and jointly. The association between gender and the survival model is shown in the joint model and not in the separate model, and this is probably due to the variable use of heath care resources, as men usually report fewer claims than women.

The mutual health insurance sector, which stores large portfolios of individual policyholders monitored over long periods of time, provides a benchmark example of the potential applications of joint modelling techniques that should broaden our understanding of the mechanisms underpinning health progression trends. Indeed, insights into the underlying trends at this individualized level should enable professionals to provide more accurate service demand predictions and to adjust the risk of mortality to better capture the baseline factors of age and gender. To date, joint modelling techniques have usually been used with quite small datasets (in the order of a few hundred individuals) and it is not easy to find applications to larger sets or discussions of the computational obstacles that need to be overcome.

This study has reported the challenging task of implementing a single joint model with a large data sample (policy holders aged 65 and over) and it has demonstrated a statistically significant dependence between a subject's past medical care usage (their use of ambulance and emergency services and admissions to hospital) and their current hazard of death. While we have no information about the number of days the subjects spent in hospital or in the emergency room, nor about the condition that required their seeking healthcare, we are able to provide a personalized survival prediction. Information on routine medical visits is specifically not used here.

## Conclusion

The results reported here, as illustrated by a real case, demonstrate that likelihood inferences based on a joint procedure are efficient and less biased than those obtained from the classic separate approach. Thus, they present obvious benefits over standard survival techniques. The positive sign of the association parameter shows that relatively high cumulative demand for emergency room, hospitalization and ambulance services is simultaneously related to a deterioration in the subject's health status and, consequently, to lower probabilities of survival. Specifically, an increase in accumulated claims can be quantified as an increase in mortality risk, for an initial age and gender thus leading to personalized survival curve prediction.
